# Infantile hypertrophic pyloric stenosis: a 4-year experience from two tertiary care centres in Cameroon

**DOI:** 10.1186/s13104-018-3131-1

**Published:** 2018-01-16

**Authors:** Rene Ndongo, Paul Nkemtendong Tolefac, Faustin Félicien Mouafo Tambo, Matin Hongieh Abanda, Marcelin Ngowe Ngowe, Olivier Fola, Bonaventure Dzekem, Patrick Eroyl Weledji, Maurice Aurelien Sosso, Jacqueline Ze Minkande

**Affiliations:** 1 Yaounde Central Hospital, Yaoundé, Cameroon; 2 Faculty of Medicine and Biomedical Sciences, University of Yaoundé 1, Yaoundé, Cameroon; 3 Douala General Hospital, Douala, Cameroon; 4 Faculty of Health Sciences, University of Buea, Buea, Cameroon; 5 Clnical Research Education Networking and Consultancy, Douala, Cameroon

**Keywords:** Infantile hypertrophic pyloric stenosis, Ramstedt, Mortality, Experience, Outcome

## Abstract

**Objective:**

This study aimed to describe the clinical characteristics of patients with infantile hypertrophic stenosis, management and its outcome in two tertiary care centres in Cameroon.

**Results:**

A total of 21 patients were included from the two centres. The mean age at presentation was 5.2 ± 1.2 weeks, predominantly male with a male-to-female ratio of 4.25:1. The triad of vomiting, visible peristalsis and palpable mass was present in only 7 (33.3%) of the participants. The diagnosis was confirmed with ultrasounds in all participants. Ramstedt pyloromyotomy was done in all participants and in 9.5% of the participants it was complicated by intra-operative duodenal perforation whereas in the postoperative period the most common complications were vomiting (6, 28.6%), sepsis (2, 9.5%), and paralytic ileus (2, 9.5%). The mortality rate from the series is 9.5%. According to univariate logistic regression: severe dehydration [OR = 5.41, 95% CI = (3.11–6.97), p = 0.002], hypokalaemia [OR = 2.63, 95% CI = (1.02–5.91), p = 0.042] and surgical site infection [OR = 3.12, 95% CI (1.22–5.64), p = 0.023] were the main predictors of mortality whereas postoperative hospital length of stay > 5 days was significantly associated with surgical site infection [OR = 2.44, 95% CI = (1.12–6.44), p = 0.002] and postoperative nausea and vomiting [OR = 3.64, 95% CI = (1.18–6.64), p = 0.022].

## Introduction

Infantile hypertrophic pyloric stenosis (IHPS) describes a disorder in infancy characterised by hyperplasia of smooth muscle fibres of the pylorus leading to narrowing of the pyloric canal and gastric outlet obstruction [1]. The incidence of IHPS varies amongst different ethnic groups and races around the world [2]. IHPS occurs in about 1–4 per 1000 live births and it is more common among male infants with a male to female sex ratio estimated at 4–6:1 [3–5], and more frequent in preterm than term neonates [4, 6]. Male gender predominance and a family history of IHPS are consistently reported risk factors and suggest a genetic component to the aetiology [7].

The paucity of published data regarding IHPS in most developing countries like Cameroon, prompted the authors to analyse this problem. The study aimed to describe the experience on the management of IHPS in two tertiary hospitals Cameroon.

## Main text

### Materials and methods

#### Study design and setting

This was a retrospective cross sectional descriptive study carried out in two tertiary hospitals in Cameroon; Douala General Hospital (DGH) and Yaoundé Central Hospital (YCH) over a period of 4 years from January 2012 to December 2016 to describe the experience in the management of patients admitted to the paediatric surgical units of these tertiary institutions. DGH and YCH are two largest hospitals located in the economic capital and administrative capital of Cameroon respectively. Both hospitals have paediatric surgical units that functions 24/24 receiving patients.

#### Study population

The study population included all infants who were admitted to the surgical units of both hospitals with the diagnosis of IHPS and subsequently benefited from surgery during the study period. We excluded all patients that had an alternative intraoperative diagnosis and those with incomplete medical records. The diagnosis of IHPS was made clinically by the typical clinical presentation of non-bilious vomiting and palpable pyloric olive mass and by abdominal ultrasound. Data was collected using pre-established medical case report forms from the patient’s medical record in the paediatric surgical units and theatre of both hospitals. Over the 4 years period, 28 cases of IHPS were confirmed using abdominal ultrasound. We excluded three cases that had an alternative intraoperative diagnosis and five cases whose medical records were missing.

#### Statistical analysis

The data was collected using epi data version 3.1 then transferred to and analysed using SPSS version 20.0. Mean and standard deviations were determined for continuous variables whereas proportions and frequency tables were used to summarize categorical variables. The level of significance was considered as p < 0.05. Univariate logistic regression was used to assess factors associated with mortality and prolonged postoperative stay greater than 5 days.

#### Ethical considerations

Ethical and administrative approval was obtained from the respective hospitals before commencement of the study. The principles of ethics involving human participants were respected throughout the study. After assessing the medical files, patient’s guardians were called for signed informed consent and this was done before information was collected from the medical files of all the participants.

### Results

#### Baseline patient characteristics

Over the 4 years period, 28 cases of IHPS were confirmed using abdominal ultrasound in both hospitals. We excluded three cases that had an alternative intraoperative diagnosis and five cases whose medical records were missing. The 21 patients included were all of the black African race. They were predominantly males with a male female sex ratio of 4.25:1. The mean age at presentation was 5.2 ± 1.2 weeks with a range of 8 days to 12 weeks and most were neonates within 2–6 weeks (16, 76.2%) of life as shown on Table 1. About half (47.6%) were first born children and breast feeding was the dominant mode of feeding in 66.7% (Table 1).

**Table 1:** Baseline characteristics of the study population showing age distribution, gestational age at birth, birth weight, position in the family and feeding methods during the first 6 months after birth

Category	Characteristic	Number	Percentage
Age at presentation (weeks)	< 2	1	4.8
	2–4	9	42.9
	4–6	7	33.3
	> 6	4	19.0
Gestational age (weeks)	< 37	12	57.1
	37–42	9	42.9
Birth weight (g)	< 25.00	14	66.7
	25.00–4.000	7	33.3
Family order	First	10	47.6
	Second	3	14.3
	Third	2	9.5
	Fourth	3	14.3
	Fifth	1	4.8
	Others	2	9.5
Feeding method	Breast feeding	14	66.7
	Artificial milk	5	23.8
	Mixed feeding	2	9.5

#### Clinical presentation and diagnosis

The diagnosis was made clinically in patients who presented with a triad of non-bilious projectile vomiting (100%), visible gastric peristalsis (47.6%) and a palpable epigastric olive mass (33.3%) as presented on Table 2. This triad was present in 7 (33.3%) of the participants. In all other patients, a suspicion was made after presenting with one or more of the above symptoms inclusive of weight loss (52.4%), dehydration (42.9%), and constipation (19.0%) (Table 2). This diagnosis was confirmed in all the patients using abdominal ultrasound.

**Table 2:** Clinical characteristics at presentation showing proportion that presented with vomiting, dehydration, visible peristalsis, palpable epigastric olive mass and other symptoms

Characteristic	Number	Percentage
Vomiting	21	100.0
Constipation	4	19.0
Weight loss	11	52.4
Dehydration	9	42.9
Visible gastric peristalsis	10	47.6
Palpable olive epigastric mass	7	33.3
Other symptoms	4	19.0

**Fig. 1:**
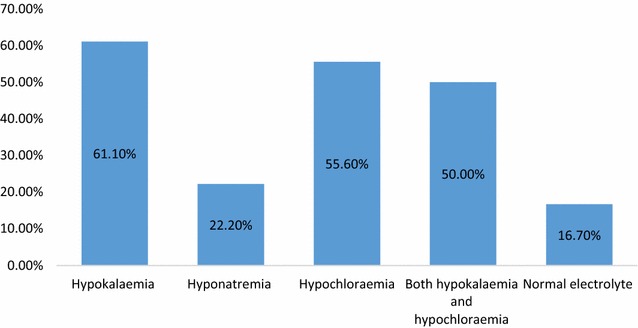
Distribution of serum electrolyte abnormalities at presentation into hypokalaemia, hyponatremia, hypochloraemia, both hypokalaemia and hypochloraemia and normal serum electrolytes

In 18 of the 21 included patients, results of serum electrolytes were available, of the available results, half had both hypokalaemia and hypochloraemia (Fig. 1), whereas 3 (16.7%) of the patients had normal serum electrolytes.

#### Management and outcome

All the patients in this study were surgically managed by Fredet-Ramstedt pyloromyotomy following resuscitation. The median preoperative hospital stay was 4.7 ± 1.1 days with a range of 2–12 days. During this preoperative period, fluid and electrolyte abnormalities, dehydration as well as anaemia were corrected prior to surgery. Fluid and electrolytes were corrected using ringers lactate and glucose 5%.

General anaesthesia was the main form of anaesthesia used in all the patients. The surgical opening was by supra-umbilical transverse incision in 17 (80.1%) of the cases while in 4 (19.9%) of the cases subcostal incision was used. There were 2 (9.5%) intra-operative mucosal (duodenal) perforations. Post-operatively, test feeds were started within 12 h of surgery in 16 (76.2%) of the infants and within 12–24 h in 3 (14.3%) participants. Two (9.5%) participants who had intraoperative perforations were kept *nil per os* for more than 24 h. A total of 13 complications were noted in 6 participants. These complications included post-operative 6 (28.6%) vomiting, 2 (9.5%) surgical site infections, 2 (9.5%) paralytic ileus (2, 9.5%) sepsis and 1 (4.8%) wound dehiscence (1, 4.8%). Two cases of death were registered in the series given a case fatality rate of 9.5%. The major causes of death were severe sepsis in the postoperative period in one case as a result of intra-operative perforation and severe dehydration and electrolyte imbalance not corrected before surgery in the other case. The mean postoperative hospital stay was 4.2 ± 1.6 days with a range of 3–12 days. According to univariate logistic regression analysis: severe dehydration on admission [OR = 5.41, 95% CI (3.11–6.97), p = 0.002], hypokalaemia on admission [OR = 2.63, 95% CI (1.02–5.91), p = 0.042] and surgical site infection [OR = 3.12, 95% CI (1.22–5.64), p = 0.023] were the main predictors of mortality whereas postoperative hospital length of stay > 5 days was significantly associated with surgical site infection [OR = 2.44, 95% CI (1.12–6.44), p = 0.002] and postoperative nausea and vomiting [OR = 3.64, 95% CI (1.18–6.64), p = 0.022].

### Discussion

Infantile hypertrophic pyloric stenosis (IHPS) was first described by Harald Hirschsprung in 1888 [8]. It is the most common cause of gastric outlet obstruction in infancy and the most common surgical emergency in a new born [9]. In this cross sectional descriptive study, the authors described their experience of the presentation and management of infantile hypertrophic pyloric stenosis in two tertiary health care centres in Cameroon. Males were predominantly affected with a male female sex ratio of 4.25:1 which is comparable to the global male female sex ratio of 4–6:1 [3]. This male predominance is equally similar to results gotten from other recent studies [7, 10]. There disorder was more common among first born infants at a rate of 47.6% compared to higher orders children. This means almost half of the cases of IHPS occur in first born children. This is similar to a study done in Ethiopia in 2014 by Tadesse and Gadisa where 56.4% occurred in first born infants [7]. The mean age of presentation of 5.2 weeks is consistent with findings in global literature [2, 11], and in findings from recent studies in Ethiopia and Tanzania [7, 10].

Infantile hypertrophic pyloric stenosis being the most common cause of gastric outlet obstruction usually presents with non-bilious vomiting [2]. This was the most predominant symptom in all the participants. This is equally similar to recent series described elsewhere in Africa where all the participants presented with vomiting [7, 10]. The classic presentation of an olive mass in the epigastric region on palpation was established only in 33.3% of the cases. This means that in about two-third of the cases of IHPS the olive is not palpable. This reduces the clinical diagnostic probability of IHPS. This low rate of palpable olive shape mass in the epigastrium is similar to 23–26% obtained in studies in Ethiopia and Tanzania [7, 10]. Preoperatively, IHPS is usually complicated by dehydration, weight loss and a characteristic hypochloraemic hypokalaemic metabolic alkalosis [12–14]. All these complications were observed in the series described with the most common electrolyte abnormality hypochloraemia and hypokalaemia. Similar electrolyte abnormalities were observed in a study in 2015 in Tanzania [10]. In a univariate analysis patients with hypokalaemia at admission were twofolds more likely to die in the postoperative period compared to others. This is similar to the Tanzanian study published in 2015 [10]. Other electrolyte abnormalities were not associated with mortality.

The gold standard in the management of IHPS remains surgery described in 1912 by Ramstedt now known as Fredet-Ramstedt extra-mucosal pyloromyotomy [1, 15]. This can be done both by traditional laparotomy or laparoscopy [16]. All our patients benefited from pyloromyotomy by laparotomy. Mucosal perforation is a rare intraoperative complication of Ramstedt’s pyloromyotomy and is indicated intraoperatively by the appearance of bilious fluid. When this occurs, repair is done by using interrupted absorbable sutures and covered with omentum [17]. In our series, intraoperative duodenal perforations were reported in 9.5% of the participants, a figure which is higher than 1–2% reported in literature [17, 18]. The rate of mucosal perforations in our series is however similar to 10.9% that was reported in a series conducted in Ethiopia by Tadesse and Gadisa [7]. Higher than expected rate of mucosal perforation in the series and other series in African Countries such as Ethiopia further explained the difficulties faced in low and middle income countries such as lack of adequately trained specialists and lack of appropriate materials. This observation calls for meticulous care to be taken when performing Ramstedt’s pyloromyotomy to prevent mucosal perforation, especially at the lower end of the incision (pyloric–duodenal junction).

Mortality after pyloromyotomy is less than 1% in most centres and when it occurs, it is usually from fluid and electrolyte depletion in infants presenting late, and inadequately corrected electrolyte problems before surgery [12]. We observed very high mortality rate of 9.5% in our series. This high mortality rate is similar to that of studies described elsewhere in Africa [7, 10]. The high mortality rate can be attributed to the high rate of intraoperative complication and postoperative infections coupled to improperly corrected fluid and electrolyte deficit before surgery. A recent study published in BMJ global health that examines the determinants of mortality in children less than 20 years after abdominal surgeries found mortality to be significantly higher (seven times) in low and middle countries (LMICs) compared to high income countries and this mortality was associated with intra-operative perforated viscus [19]. This further explains the high mortality noted in the series and related studies in Africa. The high mortality associated to our series and related studies in Ethiopia [7] and Tanzania [10] can further be explained by global shortage of surgical and anaesthetics services in these regions [20, 21]. The number of cases of IHPS that remain undiagnosed in the community may be higher than expected as most of these later die of severe dehydration and sepsis. This is especially true globally as about 5 billion people do not have access to safe and essential surgical services and in LMICs including Cameroon nine out of ten people cannot afford surgical care; only 6% of 313 million surgical procedures done to save lives are undertaken in LMICs [21–23].

### Conclusion

The series has demonstrated that infantile pyloric stenosis is 4 times more common in males compared to females and is more common among first born neonates compared to higher order neonates. The most frequent clinical presentation is non-bilious projectile vomiting. The management of choice still practiced in our setting is Fredet-Ramstedt extra-mucosal pyloromyotomy. The mortality rate is higher in this region at 9.5% and factors associated with this mortality are severe dehydration, hypokalaemia and surgical site.

## Limitations

Some limitations to our study include:

- Cross sectional nature and inability to establish spatial relationships.
- Relatively small sample size of 21 participants and short time period of 4 years.
- Solely hospital based as most patients with IHPS die in community of dehydration and electrolyte imbalance.

## Abbreviations

IHPS: infantile hypertrophic pyloric stenosis
YCH: Yaounde Cantral Hospital
DGH: Douala General Hospital
LMICs: low and middle income countries
